# The Reliability and Validity of the Malay Parent-Report Version of the Strengths and Difficulties Questionnaire

**DOI:** 10.21315/mjms2021.28.1.18

**Published:** 2021-02-24

**Authors:** Idayu Badilla Idris, Jane Barlow, Alan Dolan, Shahlan Surat

**Retraction note:**

The retraction of the manuscript titled above from the *Malaysian Journal of Medical Sciences* has been requested by the administrator of the youth *in* mind in the UK who is in charge of the SDQ questionnaire because a translation of the Malay Parent-version Questionnaire into the Malay Children-version was done and published without the necessary authorisation via the author of the manuscript, Dr Idayu Badilla Idris and co-authors.

Editor-in-Chief

Malaysian Journal of Medical Sciences

